# Birth outcomes following antiretroviral exposure during pregnancy: Initial results from a pregnancy exposure registry in South Africa

**DOI:** 10.4102/sajhivmed.v20i1.971

**Published:** 2019-09-30

**Authors:** Ushma C. Mehta, Cari van Schalkwyk, Prineetha Naidoo, Arthi Ramkissoon, Otty Mhlongo, Niren R. Maharaj, Niree Naidoo, Karen Fieggen, Michael F. Urban, Shaun Krog, Alex Welte, Mukesh Dheda, Yogan Pillay, Neil F. Moran

**Affiliations:** 1 Centre for Infectious Disease Epidemiology and Research (CIDER), School of Public Health and Family Medicine, Faculty of Health Sciences, University of Cape Town, Cape Town, South Africa; 2 South African Centre for Epidemiological Modelling and Analysis, Stellenbosch, South Africa; 3 Maternal and Adolescent Child Health Systems (MatCH), School of Public Health, University of the Witwatersrand, Johannesburg, South Africa; 4 KwaZulu-Natal Department of Health, Pietermaritzburg, South Africa; 5 Prince Mshiyeni Memorial Hospital, Durban, South Africa; 6 Division of Human Genetics, Department of Medicine, University of Cape Town, Cape Town, South Africa; 7 Division of Molecular Biology and Human Genetics, Faculty of Medicine and Health Sciences, Stellenbosch University, Cape Town, South Africa; 8 VP Health Systems, KwaZulu-Natal, Durban, South Africa; 9 Programmatic Pharmacovigilance Unit, National Department of Health, Pretoria, South Africa; 10 National Department of Health, Pretoria, South Africa

**Keywords:** pharmacovigilance, antiretrovirals, pregnancy, birth outcomes, safety, birth defect, congenital malformations, surveillance

## Abstract

**Background:**

In 2013, a pregnancy exposure registry and birth defects surveillance (PER/BDS) system was initiated in eThekwini District, KwaZulu-Natal (KZN), to assess the impact of antiretroviral treatment (ART) on birth outcomes.

**Objectives:**

At the end of the first year, we assessed the risk of major congenital malformations (CM) and other adverse birth outcomes (ABOs) detected at birth, in children born to women exposed to ART during pregnancy.

**Method:**

Data were collected from women who delivered at Prince Mshiyeni Memorial Hospital, Durban, from 07 October 2013 to 06 October 2014, using medicine exposure histories and birth outcomes from maternal interviews, clinical records and neonatal surface examination. Singleton births exposed to only one ART regimen were included in bivariable analysis for CM risk and multivariate risk analysis for ABO risk.

**Results:**

Data were collected from 10 417 women with 10 517 birth outcomes (4013 [38.5%] HIV-infected). Congenital malformations rates in births exposed to Efavirenz during the first trimester (T1) (RR 0.87 [95% CI 0.12–6.4; *p* = 0.895]) were similar to births not exposed to ART during T1. However, T1 exposure to Nevirapine was associated with the increased risk of CM (RR 9.28 [95% CI 2.3–37.9; *p* = 0.002]) when compared to the same group. Other ABOs were more frequent in the combination of HIV/ART-exposed births compared to HIV-unexposed births (29.9% vs. 26.0%, adjusted RR 1.23 [1.14–1.31; *p* < 0.001]).

**Conclusion:**

No association between T1 use of EFV-based ART regimens and CM was observed. Associations between T1 NVP-based ART regimen and CM need further investigation. HIV- and ART-exposed infants had more ABOs compared to HIV-unexposed infants.

## Introduction

Despite improvements in recent years, maternal and infant mortality rates in South Africa remain unacceptably high. HIV/AIDS (complicated by Tuberculosis and pneumonia), haemorrhage and hypertension account for more than two-thirds of the avoidable maternal deaths.^1^ The early initiation of antiretroviral therapy (ART) in pregnant women and women of child-bearing age has well-described benefits and has become standard practice in South Africa.^2^ In 2013, the World Health Organization (WHO) recommended the initiation of lifelong antiretroviral treatment for pregnant women diagnosed with HIV during pregnancy, regardless of CD4 cell count or clinical stage (Option B+).^3^ This approach was adopted by the South African National Department of Health (NDoH) in April in the same year.^4^ However, various reports have suggested that antiretroviral therapy may increase the risk of adverse birth outcomes (ABOs), including preterm delivery (PTD),^5,6^ low birth weight (LBW),^7,8^ small for gestational age (SGA),^9,10^ stillbirth (SB),^9,11^ neonatal death (NND)^9,11^ and, according to some reports, congenital malformations (CM).^12,13,14^ In most of these situations, the contribution of the underlying HIV infection and other maternal risk factors is difficult to rule out.^15^ Further studies have investigated the relative safety of different ART regimens,^16,17^ while others have assessed whether the timing of the initiation of ART, in relation to conception, influences the risk of ABOs.^6,7,8,18^

Questions persist about the risk of ABOs associated with exposure to specific antiretrovirals such as nevirapine (NVP) and protease inhibitors (PIs).^16,19^ Most recently, concerns about the safety in pregnancy of dolutegravir (DTG), a long-anticipated agent with an improved efficacy and safety profile, have been raised by early signals of higher rates of neural tube defects in infants exposed in utero at the time of conception compared to efavirenz (EFV).^12^

A robust meta-analysis of data from the outcomes of 2026 live births of women exposed to EFV during the first trimester found only one case of a neural tube defect, and the rate of CM was not higher than in infants exposed to non-EFV-containing first-trimester regimens.^20^ In preclinical primate and clinical studies, in utero exposure to tenofovir (TDF) has been associated with growth restriction, bone mineral content reduction and bone toxicity.^21,22^ However, the Antiretroviral Pregnancy Registry (APR), based in the United States, concluded that a doubling and 1.5-fold increase in risk of CMs, with EFV and TDF, respectively, could be ruled out based on the American background CM rate of 2.7%.^23^ Similarly, no increase in the risk of CM has been observed with NVP to date.^24^

Given the relative rarity of CMs, lack of available data on baseline rates of CM from low- and middle-income countries and the great number of exposures in HIV-affected settings like South Africa, Ford and others have recommended ongoing prospective birth outcomes surveillance.^20,25,26,27^ Concerns about the safety of ART in the unborn child, applicable to millions of women who are and will be receiving ART during pregnancy in the coming years, will persist in the absence of controlled studies that are able to quantify the risk of potential CMs and other ABOs such as LBW, PTD, NND, SB and SGA. Hence, in 2013, the South African NDoH implemented a pregnancy exposure registry (PER) and birth defect surveillance (BDS) system at selected sentinel sites to assess the association of medicines commonly used in pregnant women, initially focusing on antiretrovirals, with ABOs. In partnership with the KwaZulu-Natal (KZN) Provincial Department of Health and other supporting partners, the project was launched on 07 October 2013.

We describe the findings of the first 12 months of this project, focusing on the risk of CM at birth and other ABOs (pregnancy losses, NND, SGA, PTD and LBW) in infants born to mothers with confirmed exposure to specific antiretroviral regimens. Two separate analyses were conducted for CM (where the risk of teratogenic exposure is highest in the first trimester when organogenesis occurs) and for other ABOs where the risk period of exposure is less well defined and more likely to be continuous.

## Methods

### Setting

The birth defect (referred to as ‘congenital malformations’ [CM] in this article) surveillance was carried out at Prince Mshiyeni Memorial Hospital (PMMH) in the KwaZulu-Natal province of South Africa, where approximately 14 000 deliveries occur each year. HIV prevalence among pregnant women in this province at the time of the study was steadily increasing from approximately 37.4% (95% CI 35.8% – 39.0%) in 2011 to 44.4% (95% CI 42.5% – 46.3%) in 2015,^28^ with antenatal ART initiation rates increasing from 85.4% in 2013 and 2014 to 97.2% in 2015 and 2016.^29^

### Surveillance method

Five surveillance nurses surveyed the hospital’s maternity wards and labour ward registers to identify women who had recently delivered. The surveillance nurses collected information on demographics, health and health-seeking behaviour during pregnancy, obstetric and neonatal history, HIV status, medical conditions, medicine use including folate, calcium and iron supplementation, and labour/delivery information. As they only worked from Monday to Friday during office hours, there was incomplete coverage of deliveries during this initial pilot period. The women’s maternity case records (MCRs) were used to confirm and expand on what was reported during a brief interview. A systematic neonatal surface examination, recommended by the WHO, was performed on all live infants and stillbirths (whenever feasible) by the trained surveillance nurses.^30^ Birth weight, length, head circumference and reported gestational age at birth were collected from the MCR. Gestational age at birth is routinely documented by nursing staff based on the estimated date of delivery calculated during antenatal care based on the last menstrual period (LMP) reported at the first antenatal visit, and confirmed by ultrasound, when available. Major CMs identified at birth were recorded on the surveillance system’s case record form. In the case of infants in the nursery who were premature, febrile, ill, or too fragile to be unnecessarily handled, the surveillance nurses did not physically examine the infant themselves but rather referred to the clinical notes to complete the form.

In the case of major CMs, digital photographs were taken if consent was obtained from the mother. A CM confirmation form was completed and signed by a neonatologist or senior clinician in the nursery ward designated to support the surveillance system. A CM review panel comprising medical geneticists, paediatricians and neonatologists confirmed diagnoses of CMs by viewing photographs, the results of any chromosomal tests, and the assessment of the clinician at the time of the event. The panel classified CMs as major (i.e. structural changes that have significant medical, social or cosmetic consequences for the affected individual, and typically require medical intervention), or minor, and advised on inclusion in the risk factor analyses. We were guided by the example of Holmes;^31^ in that single minor anomalies, normal variations, birth marks, chromosomal or genetic anomalies/disorders, positional deformities, features of prematurity and physiological abnormalities were excluded from the analysis of teratogenic potential.

Neonates born in surrounding clinics or other health facilities but admitted to PMMH were not included in this analysis. Babies born at home, in the community or en-route to the PMMH who were brought directly to the hospital without first attending any other health facility were included. CMs diagnosed after discharge were not identified or reported in this analysis.

Miscarriages and terminations of pregnancy (TOPs) were not systematically captured, as they are not managed in the maternity wards. Complicated congenital anomalies detected on ultrasound may sometimes have been referred to Inkosi Albert Luthuli Central Hospital and delivered there, especially if neonatal surgery was anticipated. Early medical or curettage-based TOPs that did not require foeticide of the baby (i.e. before 24 weeks gestational age or a lethal foetal anomaly) would have been conducted at PMMH but might not have been captured in the maternity registers depending on the gestation.

A concurrent prospective pregnancy exposure registry project involving the recruitment of pregnant women at their first antenatal visit was implemented at 3 midwife-run obstetric units (MOUs) within the PMMH catchment area. However, as the data for this cohort were not available at the time of the analysis, we are unable to report on this prospective cohort.

### Risk analyses

We conducted two separate risk analyses for ART exposure:

- **Analysis A (see 2.3.4.)**: Risk factors for *major CM detected at birth*. As the critical exposures are likely to be during organogenesis (T1), we compared exposure ‘during the entire first trimester’ with no exposure during any part of the first trimester. Women for whom the timing of ART was uncertain in relation to the first trimester were excluded from this analysis.
- **Analysis B (see 2.3.5.)**: Risk factors for a composite of *other ABOs*, which included:
  ■ Pregnancy losses: *Birth of a dead foetus*.
  ■ Neonatal deaths (*included deaths before 28 days post-delivery which occurred prior to hospital discharge)*.
  ■ Small for gestational age (*live births with birth weight below the 10th percentile using WHO criteria*).
  ■ Preterm delivery (*live births born at gestational age less than 37 weeks*).
  ■ Low birth weight (live births born at weight < 2500 g)

As these outcomes could be linked to a broader range of exposure timings than just T1, we compared *any ART exposure* during pregnancy to a comparator group with *no HIV/ART exposure* (i.e. HIV-uninfected women).

#### Exclusions from analyses

Maternal deaths whose medical records were not accessible for review or where there was no delivery of a foetus were excluded from the analysis. Maternal deaths are systematically reviewed for causes through other national procedures.^1^ A central register of deliveries housed at the hospital was used to establish the rate of capture of deliveries through the surveillance system. Women who switched from a NVP-based regimen to an EFV-based regimen or vice versa, were excluded from all drug-specific and time-period-specific (‘entire first trimester’ vs. ‘any part of pregnancy’) analyses. Pregnancies were excluded from all risk analyses (though not from the overall demographic and ART exposure breakdown) if:

- HIV status was unknown.
- HIV status was recorded as positive but there was no record of ART (as this constitutes sub-standard care in the local context and is associated with substantially worse outcomes).
- Birth outcomes were unknown (alive or dead).
- Multiple births were recorded (as multiple births are known to have higher complication rates and higher rates of CMs).

**FIGURE 1:**
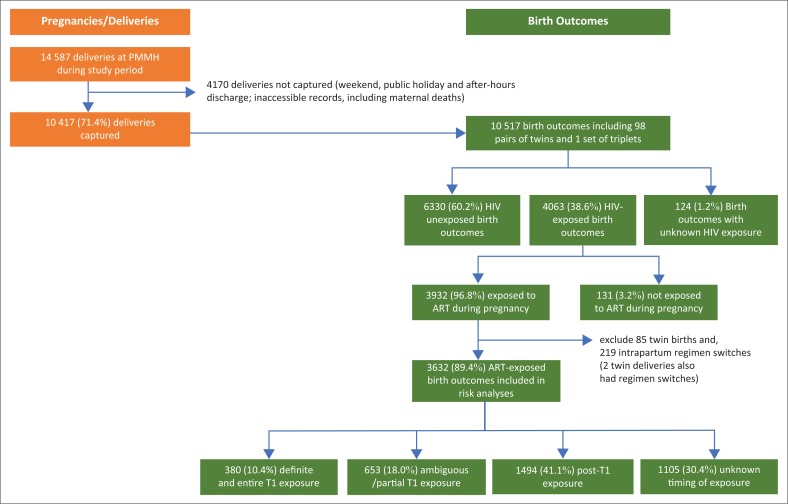
Birth outcomes by HIV/ART exposure status among pregnant women in the cohort.

Figure 1 below provides a breakdown of the cohort of pregnant women (deliveries) and their birth outcomes primarily by HIV exposure and ART exposure.

#### Antiretroviral therapy exposures

The present analysis is based on 1 year of data from an ongoing surveillance system – approximately 18 months after first-line therapy was being changed from TDF/lamivudine (3TC)/NVP to TDF/emtricitabine (FTC)/EFV. Exposures to (second-line) PIs, as well as non-standard regimens, were limited. Given the *a priori* concern about non-nucleoside reverse transcriptase inhibitors (NNRTIs), risk factor analysis was confined to assessing the effects of (1) any ART regimen, (2) EFV-based regimens and (3) NVP-based regimens.

To verify the timings of exposures reported in the MCR, women were also explicitly asked, after delivery, whether ART had been initiated ‘before’ or ‘during’ the pregnancy.

#### Risk analysis A – Major congenital malformations detected at birth

For consideration of major CM, risk factor timing is critical because the period of pregnancy when maternal drug exposure has the greatest teratogenic potential is the embryonic phase during which organ differentiation is completed. In addition, this analysis of 1 year of data was not adequately powered to conduct finely differentiated pregnancy-stage-specific analyses, and the routinely collected data on LMP, gestational age at birth and the timing of treatment initiation were often missing or poorly captured and could lead to misclassification of exposure timing. Hence, we prefer a conservative approach of only classifying birth outcomes as exposed if ART was initiated at least 2 weeks before LMP. However, as a significant number of ART initiations appear to be during the first trimester, we also considered a broader exposure category in which ART initiations within 15 weeks post LMP are included. These exposed groups were compared to an unexposed group defined as all HIV-unexposed births as well those exposed to *HIV and* ART beginning more than 15 weeks after LMP (see Table 3).

#### Risk analysis B – Composite of other adverse birth outcomes

In the risk analysis for the composite endpoint of ABOs, known singleton birth outcomes among HIV-uninfected women were used as the comparator group. Studies about the effect of ART initiation in relation to the pregnancy have elicited conflicting findings on birth outcomes.^5,6,7,17,32^ Therefore, exposure groups assessed included any ART, EFV-based and NVP-based regimens initiated a) at any time before birth; b) before pregnancy (defined conservatively as 2 weeks before LMP); and c) after onset of pregnancy (defined as LMP plus 2 weeks). A 2-week exclusion window period around LMP was used to reduce the likelihood of misclassification in this analysis.

### Statistical analyses

Data were analysed with STATA 12 (StataCorp LP, College Station, TX, USA). For Analysis B, covariates included (in a multivariable binomial regression) included maternal age category (< 18, 18–35 or > 35 years), parity (0 = nulliparous; 1 = parous) and educational completion (none or primary vs. secondary or higher). Multivariable risk factor analysis was deemed inappropriate for Analysis A given the limited number of CMs detected. A *p*-value of < 0.05, conducted using a 2-sided *t*-test, was deemed to indicate statistical significance.

### Ethical considerations

Approval for this surveillance system was obtained from the South African Medical Research Council’s ethics committee (protocol EC015-7/2013 approved on 30 July 2013). As the PER/BDS system is mooted to become a national sentinel surveillance system, consent for routine data gathering was not required, but the digital photography of infants with CMs, as well as stillbirths, which is not routine, required explicit consent.

## Results

Over 12 months (07 October 2013 to 06 October 2014), 10 417 (71.4%) of a total of 14 587 deliveries at PMMH were captured representing 10 517 birth outcomes, including 98 twin births and 1 triplet birth.

### Maternal demographics

Table 1 provides a summary of maternal characteristics, stratified by HIV status. Of the 27 maternal deaths reported during the study period, 12 (44.4%) died prior to childbirth. Only 2 of the remaining 15 (13.3%) maternal deaths were captured by the surveillance team during the reporting year due to restricted access to these medical records at the time of data collection.

**TABLE 1:** Maternal characteristics by HIV infection status.

Variable	Total			HIV uninfected			HIV infected			HIV status unknown			HIV status association *p*‡
	*N*	%	IQR†	*N*	%	IQR†	*N*	%	IQR†	*N*	%	IQR†	
Number of mothers	10 417	-	-	6280	60.3	-	4013	38.5	-	124	1.2	-	-
**Maternal age**													< 0.001
< 18 years	704	6.8	-	620	9.9	-	73	1.8	-	11	8.9	-	-
18–35 years	8596	82.5	-	5197	82.8	-	3299	82.2	-	100	80.6	-	-
> 35 years	1106	10.6	-	463	7.4	-	640	15.9	-	3	2.4	-	-
Unknown	11	0.1	-	0	0	-	1	0.0	-	10	8.1	-	-
Median	25	-	21–30	23	-	20–27	28	-	23–32	22	-	19–26	< 0.001
**Education**													0.214
None/primary	808	7.76	-	471	7.5	-	328	8.2	-	9	7.3	-	-
Secondary/Tertiary	9538	91.6	-	5773	91.9	-	3665	91.3	-	97	78.2	-	-
Unknown	71	0.7	-	33	0.5	-	20	0.5	-	18	14.5	-	-
Currently employed	1393	13.4	-	696	11.1	-	689	17.2	-	8	6.5		< 0.001
**Gravidity**													< 0.001
1	4060	39.0	-	3127	49.8	-	889	22.2	-	44	35.5	-	-
2	3509	33.7	-	1857	29.6	-	1610	40.1	-	42	33.9	-	-
> 3	2807	27.0	-	1283	20.4	-	1500	37.4	-	24	19.4	-	-
Unknown	41	0.4	-	13	0.2	-	14	0.4	-	14	11.3	-	-
Median	2	-	1–3	2	-	1–2	2	-	2–3	2	-	1–2	< 0.001
**Parity**													< 0.001
0	4309	41.4	-	3265	52.0	-	998	24.9	-	46	37.1	-	-
1–2	5285	50.7	-	2602	41.4	-	2613	65.1	-	60	48.4	-	-
> 2	809	7.8	-	409	6.5	-	396	9.9	-	4	3.2	-	-
Unknown	24	0.2	-	4	0.1	-	6	0.1	-	14	11.3	-	-
**Alcohol use during pregnancy**													< 0.001
Non-user	9352	89.8	-	5724	91.1	-	3542	88.3	-	86	69.4	-	-
Past user	786	7.6	-	407	6.5	-	370	9.2	-	9	7.3	-	-
User	23	0.2	-	9	0.1	-	13	0.3	-	1	0.8	-	-
Unknown	256	2.5	-	140	2.2	-	88	2.2	-	28	22.6	-	-
**Tobacco use during pregnancy**													0.003
Non-user	9766	93.75	-	593	94.4	-	3744	93.3	-	91	73.4	-	-
Past user	370	3.55	-	192	3.1	-	174	4.3	-	4	3.2	-	-
User	9	0.09	-	5	0.1	-	4	0.1	-	0	0.0	-	-
Unknown	272	2.61	-	152	2.4	-	91	2.3	-	23	4.0	-	-
**Illicit substance use during pregnancy**													0.003
Non-user	9770	93.8	-	5930	94.4	-	3748	93.4	-	92	74.2	-	-
Past user	364	3.5	-	192	3.1	-	168	4.2	-	4	3.2	-	-
User	4	0.04	-	1	0.0	-	3	0.1	-	0	0.0	-	-
Unknown	279	2.7	-	157	2.5	-	94	2.3	-	28	22.6	-	-
**Gestational age at first ANC**													< 0.001
< 12 weeks	500	4.8	-	275	4.4	-	224	5.6	-	1	0.8	-	-
12 to 20 weeks	4057	39.0	-	2415	38.5	-	1632	40.7	-	10	8.1	-	-
21 to 28 weeks	3399	32.6	-	2145	34.2	-	1240	30.9	-	14	11.3	-	-
> 28 weeks	1092	10.9	-	681	10.8	-	405	10.1	-	6	4.8	-	-
Unknown	1369	13.1	-	764	12.2	-	512	12.8	-	93	75	-	-
Median age	20	-	16-26	21	-	16-26	20	-	16-25	24	-	18-28	< 0.001
**Number of ANC visits during current pregnancy**													< 0.001
0–3 ANC visits	2658	25.52	-	1715	27.3	-	932	23.2	-	17	13.7	-	-
> 4 ANC visits	7341	70.47	-	4381	69.8	-	2944	73.4	-	16	12.9	-	-
Unknown	418	4.01	-	190	3.0	-	137	3.4	-	91	73.4	-	-
Median	5	-	3-6	5	-	3-6	5	-	4-7	3	-	2-6	< 0.001
Reported previous adverse pregnancy outcomes (*N* = 6316)§	344	5.4	-	151	2.4	-	192	4.8	-	1	0.8	-	0.05
**Medical conditions**													
Pre-existing diabetes	34	0.3	-	26	0.4	-	8	0.2	-	0	0.0	-	0.077
Epilepsy	53	0.5	-	28	0.4	-	24	0.6	-	1	0.8	-	0.217
Tuberculosis (TB)	202	1.9	-	53	0.8	-	148	3.7	-	1	0.8	-	< 0.001
Syphilis	177	1.7	-	81	1.3	-	96	2.4	-	0	0.0	-	< 0.001

† , IQR – Interquartile Range; ANC – Antenatal Care.

‡ , The hypothesis in each case is that there is no association between known HIV-status and the characteristic described. At a 5% level of significance, a *p*-value of less than 0.05 can be interpreted as a significant association between HIV status and the given characteristic.

§ , Analysis excludes primagravidae.

Most of the 10 417 women (10 293, 98.8%) had a known HIV status, of whom 4013 (38.5%) were HIV-positive. The HIV-positive women had 4063 babies (live or stillborn). HIV-infected women tended to be older than HIV-uninfected women; teenage pregnancies in the HIV-uninfected group were 5 times more common than the HIV-infected cohort (9.9% vs. 1.8%). Self-reported use of alcohol, tobacco and illicit substances was higher among HIV-infected women.

Only 195 women (1.9%) did not seek any antenatal care before delivery. More than two-thirds (70.5%) of the cohort had four or more antenatal visits. However, almost half (43.1%) first sought care after 20 weeks of gestation. The prevalence of previous ABOs was reported for multigravidae (*n* = 6316) with only six women (0.1%) reporting previously giving birth to infants with CMs.

HIV prevalence increased with age, reaching 58.3% (640/1106) in women over 35 years of age. The prevalence in multigravidae (49.2%) was more than twice that in primigravid women (21.9%). Women who reported being currently employed had a higher prevalence of HIV than unemployed women. Both TB and syphilis were more commonly reported in HIV-infected women.

### Birth outcomes

Among the birth outcomes reflected in Table 2, 10 197 were live births, 275 (2.6%) pregnancy losses, 85 (0.8%) NND, 852 (8.1%) SGA and 56 (0.5%) new-borns with CMs detected at birth (Supplementary Table 1). Thirty-seven of the CMs were eligible for inclusion in the teratogenicity analysis (Analysis A), of which 11 (29.7%) occurred in women on ART during pregnancy (described in Table 4). The other 19 CMs were excluded from the analysis based on Holmes’s criteria noted earlier. Extra two CMs were excluded from the risk analysis as they occurred among women whose ART exposure time was uncertain or who switched ART regimens during critical or uncertain times.

**TABLE 2:** Birth outcome by HIV infection status.

Birth outcome	Total		HIV negative		HIV positive		HIV status unknown		*p*†
	*N*	%	*N*	%	*N*	%	*N*	%	
Number of birth outcomes‡	10 517	-	6330	60.2	4063	38.6	124	1.2	-
Live births (incl. NND)	10 197	97.0	6162	97.3	3941	97.0	94	75.8	0.249
Pregnancy losses (Stillbirths + Abortions)	275	2.6	149	2.4	109	2.7	17	13.7	0.081
Neonatal Death (NND)	85	0.8	44	0.7	39	1.0	2	2.4	0.139
Congenital malformations	56	0.5	36	0.6	20	0.5	0	0.0	0.603
Congenital malformations (included only)§	37	0.4	26	0.4	11	0.3	0	0.0	0.242
Unknown birth outcome	45	0.4	10	0.3	13	0.3	13	10.5	-
Birth weight in kg (Mean + s.d.)	3.0	0.6	3.0	0.6	3.0	0.7	2.7	0.9	< 0.001
Small for gestational age (SGA) (< 10th percentile)	852	8.1	482	7.6	364	9.0	6	4.8	0.011
Low birth weight (LBW) (< 2500 g)	1287	12.2	685	10.8	587	14.4	15	12.1	< 0.001
Birth weight unknown	34	0.3	18	0.3	15	0.4	1	0.8	-
Twins/ triplets (1 set)	199	1.9	99	1.6	100	2.5	0	0.0	0.001
Male	5288	50.3	3191	50.4	2043	50.3	54	43.5	0.383
Gestational age at birth	-	-	-	-	-	-	-	-	< 0.001
Preterm < 37 weeks	2285	21.7	1281	20.2	970	23.9	34	27.4	-
Term delivery 37–40 weeks	7578	72.1	4652	73.5	2874	70.7	52	41.9	-
Post term > 40 weeks	488	4.6	326	5.2	160	3.9	2	1.6	-
Unknown	166	1.6	71	1.1	59	1.5	36	29.0	-

† , The hypothesis in each case is that there is no association between known HIV-status and the characteristic described. At a 5% level of significance, a *p*-value of less than 0.05 can be interpreted as a significant association between HIV status and the given characteristic.

‡ , Denominator includes 98 twins and 1 set of triplets = 100 more infants than number of pregnant women.

§ , Included cases are congenital malformations that could be influenced by environmental exposures and therefore included in teratogenicity analysis. Decision on whether or not to include cases are made by the congenital malformation review panel.

Of the 3632 ART-exposed singleton births, the vast majority of births (3391, 93.4%) reported being on the recommended first-line adult ART regimen, TDF-FTC-EFV. There were only 96 singleton births (2.6%) exposed to NVP-based regimens during pregnancy, at least 58 (60.4%) of whom were already on treatment before pregnancy. In total, there were 3465 (95.4%) births exposed to an EFV-based ART regimen, of which 306 (8.8%) were initiated before the pregnancy and an additional 641 (18.5%) were initiated during the first trimester.

### Antiretroviral therapy during pregnancy

Table 3 describes the in-utero antiretroviral exposures during the course of pregnancy. 3932 infants were exposed to ART during pregnancy (96.8% of HIV-exposed infants). Twin births and births exposed to more than one ART regimen during pregnancy were excluded from the risk analysis. Uncertain timing of exposures due to the uncertainty of the temporal ordering of LMP and ART initiation resulted in almost a third (30.4%) of all singleton births being excluded from analysis (1105 of 3632). Of the remaining births, 380 (10.4%) were exposed from conception, and 653 (18.0%) initiated treatment some time during the first trimester. In 56 cases, ART exposure was classified as having occurred prior to pregnancy based only on self-reported timing in the absence of a reported LMP.

**TABLE 3:** Births with antiretroviral therapy exposure during pregnancy in HIV infected women.

Variable	Total ever exposed in utero		Definitive and entire first trimester exposure		Ambiguous/partial first trimester exposure		First trimester non-exposure		Unknown/uncertain exposure time (Excluded)		Self-reported as ‘Before pregnancy’ without exposure dates	
	*N*	%*	*N*	%†	*N*	%‡	*N*	%§	*N*	%¶	*N*	%††
Total births in HIV-infected women	4063	-	-	-	-	-	-	-	-	-	-	-
No ART exposure reported	131	3.2	-	-	-	-	-	-	-	-	-	-
Number exposed to ART regimen	3932*	96.8	486	12.4	667	17.0	1541	39.2	1238	31.5	74	1.9
ART regimens switched during pregnancy	219	5.4‡‡	-	-	-	-	-	-	-	-	-	-
Twin babies exposed to ART regimen	85	2.1	-	-	-	-	-	-	-	-	-	-
Number exposed to ART regimen (excluding those who switched and excluding twin births)	3632	89.4	380	10.4	653	18.0	1494	41.1	1105	30.4	56	1.5
Regimens (excluding those who switched and twin births)
TDF/FTC/EFV	3391	-	261	7.7	641	18.9	1473	43.4	1016	30.0	43	1.3
TDF/3TC/EFV	57	-	30	52.6	0	0.0	3	5.3	24	42.1	1	1.8
TDF/FTC/NVP	75	-	45	60.0	4	5.3	5	6.7	21	29.0	3	4.0
D4T/3TC/NVP	8	-	5	62.5	0	0.0	0	0.0	3	37.5	1	12.5
Other EFV-based regimens**	17	-	15	88.2	0	0.0	1	5.9	1	5.9	3	17.6
Other NVP-based regimens***	13	-	8	61.5	1	7.7	0	0.0	4	30.8	2	15.4
PI-based regimens****	30	-	7	46.7	4	13.3	4	13.3	15	50.0	0	0.0
Missing/Unknown regimens	41	-	9	22.0	3	7.3	8	19.5	21	51.2	3	7.3

† , ART initiated before reported LMP and continued on the specific regimen throughout the first 15 weeks of pregnancy; in the absence of known LMP, ART initiated at least 12 months before delivery or in the absence of any dates, exposure self-reported by the woman as being initiated before pregnancy (i.e. last column: ‘Self-reported as ‘Before pregnancy’ without exposure dates’).

‡ , Cases excluded from birth risk analysis because date of ART onset was reported to be between 2 weeks prior to LMP and LMP+15 weeks.

§ , First trimester unexposed birth outcomes where ART was initiated at least 15 weeks after first date of the last menstrual period.

¶ , Cases excluded: where date of ART onset was unknown; LMP date was unknown (and ART onset was reported as < 12 months prior to infant’s date of birth) or where there was discordance between reported dates of exposure and woman’s reporting of whether treatment was initiated before or during the pregnancy.

†† , Cases that were classified as ‘Exposed’ based on the woman’s self-reported timing of ART exposure as being ‘before pregnancy’ but where LMP or onset date was not reported. These cases are included as Definitive and entire first trimester exposures.

‡‡ , Regimen was switched in 2 twin pregnancies.

* , Of 4063 HIV-exposed births, 3932 were exposed to ART in utero;

** D4T/3TC/EFV or AZT/3TC/EFV;

*** AZT/3TC/NVP or NVP/3TC/TDF;

**** TDF/3TC/LPV/r; TDF/FTC/LPV/r; AZT/3TC/LPV/r; TDF/3TC/ATV/r; AZT/3TC/ATV/r; TDF/FTC/ATV/r.

### Congenital malformations at birth among HIV-exposed birth outcomes

HIV infection in the mothers was reported in 11 of the 37 (29.7%) included CM cases (Table 2), all of whom received ART at some time during pregnancy as described in Table 4. Five of these infants were exposed to ART continuously from conception throughout T1. One of these had a regimen change to TDF/FTC/EFV during the second trimester of pregnancy; two were initiated on ART at some time during the first trimester, and four were initiated during the second trimester.

**TABLE 4:** Summary of congenital malformations reported in HIV-infected women on antiretroviral treatment.

Number	ART regimen exposure/s	Onset of ART regimen in relation to LMP†	Congenital malformations
**Definitive and Entire First Trimester Exposure**			
1‡	D4T/3TC/NVP	5 years pre-LMP	Microcephaly, flat nasal bridge, short neck, left club foot
2	TDF/3TC/NVP	3 years pre-LMP	Hydrocephalus
3	TDF/3TC/EFV	4.3 years pre-LMP	Unilateral hypoplastic thumb
4§	TDF/FTC/EFV	Self-reported as starting before pregnancy but start date recorded as 6 months post LMP	Talipes equinovarus, and low set ears
**Ambiguous/Partial Exposure – First Trimester Initiation**			
5¶	TDF/3TC/NVP - switched to TDF/FTC/EFV in second trimester	Initiated NVP-based regimen in first trimester	Lumbar Myelomeningocele
6††	TDF/FTC/EFV	57 days post-LMP	Myelomeningocele
7	TDF/FTC/EFV	12 weeks post-LMP	Cleft Lip and Palate
**Second Trimester ART Initiation**			
8‡‡	AZT/3TC/LPV/r	4 months post-LMP	Hypospadias
9	TDF/FTC/EFV	4 months post-LMP	Dysmorphic facial features, micrognathia, unilateral preaxial polydactyly
10	TDF/FTC/EFV	4 months post-LMP	Exomphalos
11	TDF/FTC/EFV	6 months post-LMP	Hypospadias

ART, antiretroviral therapy; LMP, last menstrual period; NVP, Nevirapine.

† , LMP = First date of last menstrual period.

‡ , Mother reported use of alcohol before pregnancy.

§ , Case excluded from risk analysis due to uncertain timing of initiation in relation to pregnancy.

¶ , Neonatal death post discharge.

†† , Baby born at 3100 g 2 months before the estimated due date suggesting inaccurate LMP.

‡‡ , Unusual regimen as mother was part of a clinical trial (no record of prior exposures to first line treatments).

In addition to the 2 neural tube defects reported among infants of the women on ART, a case of anencephaly was reported in an HIV-unexposed infant.

### Risk analyses

The numbers in Table 3 may not directly match with those in Tables 5 and 6 because cases with missing values for the outcomes concerned were excluded from the denominators in Tables 5 and 6.

**TABLE 5:** Risk of congenital malformations detected at birth: Analysis A.

Variable	Number of CM		*N*	Unadjusted Risk ratio		*p*
	*N*	%		Risk ratio	95% CI	
**Primary risk analysis**						
Risk in HIV-negative pregnancies + HIV-positive late initiation pregnancies	29	0.4	7532	1.00	-	-
ART initiated before pregnancy	3	0.8	368	2.11	0.65–6.92	0.214
EFV-based regimen initiated before pregnancy	1	0.3	297	0.87	0.12–6.40	0.895
NVP-based regimen initiated before pregnancy	2	3.5	56	9.28	2.27–37.94	0.002
**Sensitivity Analysis (including initiation during first 15 weeks post LMP)**						
ART initiated before LMP + during T1	6	0.6	998	1.56	0.65–3.75	0.319
EFV-based regimen initiated before LMP + during T1	3	0.3	915	0.85	0.26–2.79	0.791
NVP-based regimen initiated before LMP + during T1	2	3.2	61	8.52	2.08–34.90	0.003

NVP, Nevirapine; LMP, last menstrual period; CI, confidence interval.

Risk Analysis B, comparing the risk of the composite endpoint of other ABOs with ART initiation (1) at any time during pregnancy, (2) before and (3) during pregnancy is reflected in Table 6.

**TABLE 6:** Risk of other adverse birth outcomes:† Analysis B.

Risk analyses	Number of ABOs		*N*	Unadjusted risk ratio		*p*	Adjusted risk ratio		*p*
	*N*	%		Risk ratio	95% CI		Risk ratio	95% CI	
Singleton births to HIV negative women	1597	26.0	6134	1.00					
ART initiated any time during or before pregnancy	1069	29.9	3577	1.15	1.08–1.23	< 0.001	1.23	1.14–1.31	< 0.001
ART initiated before conception	118	31.2	378	1.20	1.03–1.40	0.022	1.34	1.14–1.58	0.001
ART initiated during pregnancy	617	29.2	2112	1.12	1.04–1.21	0.004	1.20	1.11–1.30	< 0.001
EFV-based regimen initiated any time during or before pregnancy	1015	29.8	3411	1.14	1.07–1.22	< 0.001	1.22	1.14–1.31	< 0.001
EFV-based regimen initiated before conception	93	30.6	304	1.18	0.99–1.40	0.07	1.31	1.09–1.57	0.004
EFV-based regimen initiated during pregnancy	608	29.2	2083	1.12	1.04–1.21	0.005	1.20	1.11–1.30	< 0.001
NVP-based regimen initiated any time during or before pregnancy	32	33.3	96	1.28	0.96–1.70	0.09	1.46	1.10–1.95	0.01
NVP-based regimen initiated before conception	20	34.5	58	1.32	0.93–1.89	0.123	1.54	1.07–2.20	0.019
NVP-based regimen initiated during pregnancy	3	30.0	10	1.15	0.45–2.97	0.769	1.29	0.50–3.32	0.6
ART initiated before conception	118	31.2	378	1.00	-	-	-	-	-
ART initiated during pregnancy	617	29.2	2112	0.94	0.79–1.10	0.427	0.90	0.76–1.07	0.237
EFV-based regimen initiated before conception	93	30.6	304	1.00	-	-	-	-	-
EFV-based regimen initiated during pregnancy	608	29.2	2083	0.95	0.80–1.14	0.613	0.93	0.77–1.12	0.42
NVP-based regimen initiated before conception	20	34.5	58	1.00	-	-	-	-	-
NVP-based regimen initiated during pregnancy	3	30.0	10	0.87	0.32–2.39	0.787	0.57	0.17–1.98	0.379
EFV-based regimen initiated before conception	93	30.6	304	1.00	-	-	-	-	-
NVP-based regimen initiated before conception	20	34.5	58	0.89	0.60–1.31	0.551	0.80	0.54–1.19	0.267

ABOs, adverse birth outcomes; ART, antiretroviral treatment; EFV, Efavirenz; NVP, Nevirapine; CI, confidence interval.

† , Other ABOs include pregnancy losses, early neonatal deaths, preterm delivery and small for gestational age.

There was no significant difference in risk of CM in births exposed to ART during the first trimester compared to HIV-unexposed births and HIV-exposed births only exposed to ART beyond T1 (2.11 [95% CI 0.65–6.92; *p* = 0.214]; Table 5).

The first-trimester exposure to the EFV-based treatment (0.87 [95% CI 0.12–6.40]) was not associated with an increased risk compared to births not exposed to ART in the first trimester. However, there was a higher risk of CMs in births with pre-pregnancy initiation of NVP-based ART (9.28 [95% CI 2.27–37.94; *p* = 0.002]) compared to births not exposed to ART in the first trimester. Similar findings were obtained when a sensitivity analysis was conducted including cases with first-trimester ART initiation.

After adjusting for age category, parity and education, ART and EFV-based ART initiation regardless of the timing was associated with a higher rate of ABOs compared to HIV-uninfected women (adjusted: 1.23 95% CI 1.23–1.31; *p* < 0.001; 1.22 95% CI 1.14–1.31; *p* = 0.001). There was a tendency towards higher risk ratios when ART-, EFV- and NVP-based regimens were initiated before conception compared to initiation after conception (Table 6).

However, no difference in ABOs was noted when comparing ART initiation before pregnancy against ART initiated after conception 0.90 (95% CI 0.76–1.07; *p* = 0.237). This was also the case when the timing of the initiation of EFV- and NVP-based regimens was assessed (EFV 0.93 (95% CI 0.77–1.12; *p* = 0.42; NVP 0.57 (95% CI 0.17–1.98; *p* = 0.379). Moreover, there was no significant difference between the risk of ABOs when comparing pre-pregnancy initiation of EFV-based ART with pre-pregnancy initiation of NVP-based ART (adjusted risk ratio: 0.80 95% CI 0.54–1.19; *p* = 0.267).

## Discussion

These data represent the findings of the first year of a drug safety surveillance system aimed at improving our understanding of the safety of medicines commonly used by both HIV-infected and HIV-uninfected pregnant women in South Africa. A substantial proportion of the women (4013, 38.5%) were HIV-infected, the majority of whom were on ART (3932, 96.8%) and of whom 3467 (93.4%) were receiving the recommended first-line regimen of TDF/FTC/EFV according to SA NDoH guidelines. Only 96 women were on a NVP-based regimen during the pregnancy, mostly initiated prior to conception (Table 3). The very small number of women (40) receiving PI-containing regimens precludes the assessment of their association with primary outcomes.

Risk analysis was conducted only on the subset of CM (‘included CM’) which could plausibly have been caused by teratogenic exposures, as assessed by clinical geneticists using predefined criteria.^31^ Reassuringly, and in line with previous studies and review,^16,17,22,23,24,33^ we did not find an increased risk of CM among infants exposed to EFV in the first trimester. The two CMs reported (hypoplastic thumb and club foot) in two infants with first trimester exposure are unlike previously reported CM associated with EFV.^13,14^ It is unlikely that the reported myelomeningocele (a neural tube defect) in an infant exposed to TDF/FTC/EFV later in the first trimester was causally linked to the treatment given during the late onset of treatment initiation in the pregnancy.^34^

There was a significant statistical association between first trimester exposure to NVP-based regimens and the occurrence of congenital malformations. This statistical association will require accrual of further evidence over time (preferably prospectively collected data). Note: (1) the lack of a postulated common mechanism for these defects; (2) the small number of cases (3 cases) and (3) potential confounders such as maternal age, underlying disease severity, nutritional state, evidence of alcohol and illicit substance abuse and other potential teratogenic exposures which should be explored.

Our findings of an increased risk of other ABOs in HIV-exposed infants compared to HIV-unexposed infants assessed in Analysis B (Table 6) are well described.^6,7,10,16,17,35^ Given the exclusion of HIV-positive untreated pregnancies (noted above as representative of substandard access to care and a particularly vulnerable population), it is not possible to make a direct comparison between treated and untreated HIV-positive mothers. As there is no particular suspicion of association between HIV status and CMs, this is probably not a major limitation of the ‘Analysis A’. For ‘Analysis B’ which deals with composite ABOs, this shows that differences observed between HIV-exposed and HIV-unexposed birth outcomes are composed of mitigation of HIV infection by ART, possible adverse effects of ART and residual confounding. The effect size appeared to be bigger with NVP-based regimens than with EFV-based regimens, although a head-to-head comparison between EFV-based and NVP-based regimens in the first trimester yielded no significant difference in ABOs. Our findings of the lack of effect of timing of exposure on ABOs as a composite endpoint supports similar findings by Malaba^6^ and others.^7,32^

Due to pragmatic challenges with implementation during the first year of surveillance, coverage rates for neonatal death (53.1%) at the hospital were found to be lower than the total coverage for all deliveries (71.4%), suggesting an under-representation of these outcomes and a diminished chance of assessing risk factor associations.

The overall detection rate for CMs identifiable by surface examination was lower (0.5%) compared to 0.67% reported in other LMIC settings^35^ as only malformations identified through a surface examination conducted at the time of birth were identified and included in the analysis. The incomplete coverage of stillbirths that were cremated or taken home by the family prior to a surface examination being performed by the surveillance nurses could have contributed to under-detection, as well as exclusion of serious CMs requiring urgent referral to other institutions, either prenatally for termination or delivery or postnatally for additional care. Medical termination of pregnancies, miscarriages and early stillbirths were also not captured in this cohort, as these admissions were not seen in the labour wards.

We have no evidence to suggest that the HIV status of the women influenced the coverage in a way that would introduce a bias into the analysis, although it is possible that the under-ascertainment of CMs would make detection of an increased birth prevalence of CMs related to ART more difficult. Improved capacity for the detection and assessment of CMs, including the capture of terminations of pregnancies, was identified as a priority measure for refining the surveillance system at the site.

The surveillance programme was initiated approximately 18 months after NVP-based first-line regimen was replaced by the EFV-based regimen. Therefore, the cohort of women on NVP-based treatment had been initiated on treatment before the new guidelines were implemented and, therefore, probably on ART treatment for longer than those on the EFV-based regimens. As most women have likely been switched from NVP-based regimens to an EFV-based regimen as the cohort expands, it is unlikely that South African sites alone will generate adequate first-trimester NVP exposures to further explore this association. Previous international reports have not found an association between NVP-based ART exposure and the risk of CMs but with other ABOs such as stillbirths.^16,36,37^

Data on early exposure to TDF, FTC and EFV will dominate future analyses, facilitating improved quantification of risks of ABOs. Similarly, information on the safety of second-line regimens will grow. The recent safety signal of an association between dolutegravir-based ART and the risk of neural tube defects in Botswana is already delaying the use of this effective regimen in women with child-bearing potential. This has highlighted the importance of ongoing pregnancy exposure surveillance in African settings where the burden of HIV and other communicable diseases is high, given a growing pipeline of new medicines, including vaccines, targeting pregnant women.

The conservative inclusion of only cases with a definite timing of exposure into the risk factor analysis meant that a significant proportion (30.4%) of the cohort of ART-exposed birth outcomes were excluded from the analyses. However, we found no difference between the cohort that was included in the risk analysis and the excluded group in terms of age category, parity and educational status, and believe that the exclusion of these cases is unlikely to bias our findings. As the surveillance system matures, we also expect improved data on ART exposures from record linkage between maternal and child health services and the data captured in the national electronic ART registration system (Tier.Net). Reported LMP dates will be augmented with data on gestational age from routine early ultrasound scans to better characterise the timing of exposures. Nevertheless, the low rates of SGA and PTD reported in this study suggest that the estimations of gestational age at birth may not be accurate. This can be attributed to the low reporting of LMP, late presentation of prenatal ultrasound and lack of a standardised assessment used for assessing prematurity at birth in labour ward. In future analyses, low birth weight, albeit crude, may be a more reliable endpoint to measure instead of SGA and PTD.

The background rates of common malformations detectable at birth will be more reliably observed as capacity development activities at this sentinel surveillance site are expanded, including systematic assessment of stillbirths and improved access to clinical genetic expertise at the site for better characterisation and diagnosis of CMs detected at the time of birth. The surveillance system is currently only able to detect external visible malformations detectable immediately after delivery. As the surveillance system matures and the ABOs under scrutiny accumulate, multivariable analyses incorporating the data collected on other risk factors and exposures, including the use of herbal and traditional medicines, illicit drugs, tobacco and alcohol, can be incorporated into the risk analyses as can calendar time.

## Conclusion

We found no association between first-trimester exposure to EFV-based treatments and risk of congenital malformations. The risk of other ABOs is greater among HIV-exposed versus HIV-unexposed infants. However, the timing of initiation of EFV and NVP-based ART regimens, in relation to conception, did not affect the risk of ABOs. While reassuring, the data are not yet sufficient to completely exclude relevant drug-related risks associated with the components of the current first-line ART regimen for pregnant women in South Africa, and surveillance should be continued. The unexpected statistically significant associations, found between T1 exposure to NVP and CMs, demonstrate the need to continue to monitor the safety of medicines in pregnancy even in the absence of previous animal and human evidence of teratogenic risk. These initial signals of statistical association need to be reassessed using multivariable analysis as the surveillance system expands. The overall low rate of CMs in women on the current first-line ART regimen (TDF/FTC/EFV) for pregnant women in South Africa is reassuring and supported by similar work in Botswana.^12,16,17,38^

This PER/BDS surveillance system, having produced significant unexpected, albeit crude, associations in its first year, is clearly creating a rich resource of data. This will be critical for monitoring potential risk, for mothers and their infants, for widely used medicines and for addressing potential concerns with formal measures of low risks.
